# MRCK as a Potential Target for Claudin-Low Subtype of Breast Cancer

**DOI:** 10.7150/ijbs.88285

**Published:** 2024-01-01

**Authors:** Hirohito Yamaguchi, Ling-Chu Chang, Olin Shih-Shin Chang, Yu-Fu Chen, Yu-Chun Hsiao, Chen-Shiou Wu, Mien-Chie Hung

**Affiliations:** 1 Graduate Institute of Biomedical Sciences, China Medical University, Taichung City 406040, Taiwan R.O.C.; 2 Center for Molecular Medicine, China Medical University Hospital, Taichung City 40402, Taiwan R.O.C.; 3 Research Center for Cancer Biology, China Medical University, Taichung City 40402, Taiwan R.O.C.; 4 Department of Molecular and Cellular Oncology, University of Texas MD Anderson Cancer Center, Houston, TX 77030, USA.; 5 Bristol-Myers Squibb, Redwood City, CA 94063, USA.

**Keywords:** TNBC, Claudin-low, MRCK, YAP/TAZ, Focal adhesions, p65

## Abstract

To find new molecular targets for triple negative breast cancer (TNBC), we analyzed a large-scale drug screening dataset based on breast cancer subtypes. We discovered that BDP-9066, a specific MRCK inhibitor (MRCKi), may be an effective drug against TNBC. After confirming the efficacy and specificity of BDP-9066 against TNBC *in vitro* and *in vivo*, we further analyzed the underlying mechanism of specific activity of BDP-9066 against TNBC. Comparing the transcriptome of BDP-9066-sensitive and -resistant cells, the activation of the focal adhesion and YAP/TAZ pathway were found to play an important role in the sensitive cells. Furthermore, YAP/TAZ is indeed repressed by BDP-9066 in the sensitive cells, and active form of YAP suppresses the effects of BDP-9066. YAP/TAZ expression and activity are high in TNBC, especially the Claudin-low subtype, consistent with the expression of focal adhesion-related genes. Interestingly, NF-κB functions downstream of YAP/TAZ in TNBC cells and is suppressed by BDP-9066. Furthermore, the PI3 kinase pathway adversely affected the effects of BDP-9066 and that alpelisib, a PI3 kinase inhibitor, synergistically increased the effects of BDP-9066, in *PIK3CA* mutant TNBC cells. Taken together, we have shown for the first time that MRCKi can be new drugs against TNBC, particularly the Claudin-low subtype.

## Introduction

Breast cancer is classified into several subtypes according to the expression of proteins such as hormone receptors and HER2, and is a highly heterogeneous cancer with different etiologies, therapeutic effects, and prognoses 1. Among them, triple-negative breast cancer (TNBC), which does not express hormone receptors or HER2, is the subtype with the worst prognosis, accounting for 15-20% of all breast cancers 2. In the classification based on gene expression profiling, TNBC mainly includes the Basal and Claudin-low subtypes 3. The basal subtype breast cancer highly expresses genes specific to basal cells of normal mammary tissue, while the Claudin-low type has high expression of genes related to epithelial-mesenchymal transition (EMT), mammary stem cells/breast cancer initiating cells, and immune cell infiltration 3.

As systemic treatment for TNBC, chemotherapy is mainly used because drugs targeting estrogen receptors and HER2 cannot be used for it 4. In fact, TNBC is highly sensitive to chemotherapy, but many patients eventually become resistant to chemotherapy. An antibody against PD-1, an immune checkpoint inhibitor, was recently approved by the U.S. Food and Drug Administration (FDA) for the treatment of TNBC, but the response rate is only 20% in TNBC patients 5. About 5% of breast cancer patients have *BRCA* gene mutations, and a majority of those breast cancers are TNBC 6. In these cancers, PARP inhibitors are used as effective therapeutic agents 7. In 2020, a new antibody drug conjugate that targets Trop2, which is overexpressed in TNBC, has been approved as a second-line therapy for TNBC 8. Therefore, unlike five years ago, there are several options for non-chemotherapy systemic therapy in patients with TNBC. However, treatment options are still fewer than those of other subtypes, and metastatic TNBC eventually becomes resistant to all these drugs. Therefore, the development of more therapeutic agents for TNBC is an important issue.

Cdc42, along with Rho and Rac, is a member of the Rho GTPases family and regulates multiple signaling pathways involved in many cellular functions, including regulation of cytoskeletal dynamics, cell polarity, migration, and proliferation, and cancer progression 9. These GTPases are primarily activated by integrins, receptor tyrosine kinases (RTKs), and G-protein-coupled receptors (GPCRs), each of which activates different downstream protein kinases 9. MRCK is a protein kinase that functions downstream of Cdc42. After being activated by Cdc42, MRCK promotes actin polymerization, stress fiber formation and binding to cell membranes by phosphorylating downstream molecules such as myosin light chain 2 (MLC2), LIM domain kinase (LIMK), and myosin phosphatase target subunit-1 (MYPT1) 10.

In this study, we searched for new TNBC-specific drug targets from the unbiased drug-screening database and found that BDP-9066, a MRCK inhibitor (MRCKi), could be novel therapeutic agents for TNBC. We analyzed the mechanisms that determine the specificity of BDP-9066 for TNBC and showed that it inhibits the YAP/TAZ and NF-κB pathways, which are particularly important in TNBC. Furthermore, we showed that the BDP-9066 is most effective against Claudin-low subtype breast cancer, suggesting the potential of MRCK as a new drug target for TNBC where Claudins can be used as biomarkers.

## Materials and methods

### Database screening of effective drugs for TNBC

The Z-score of IC50 data of breast cancer cell lines for various drugs was downloaded from *Genomics of Drug Sensitivity in Cancer* (https://www.cancerrxgene.org/). The data extraction of particular drugs, statistical analyses, and graphing were performed using R's basic package and Tidyverse package (https://www.r-project.org/).

### RNA sequencing and RNA expression data analysis

BT549 cells were treated with 0.5 µM BDP-9066 for 8 and 16 hours and total RNA was isolated with TRIZOL solution (ThermoFisher Scientific, Waltham, MA, USA). Assessment of RNA quality, library construction, RNA sequencing were processed and analyzed by GENEWIZ (Burlington, MA, USA). The pipeline used for RNA sequencing data analysis was Cutadapt (data cleaning), HISAT2 (alignment), and HTSeq (counting). Differentially expressed genes were analyzed using DESeq2. The pathway analysis was performed by using g: Profiler 11. The dot plot charts were created using Ggplot2 (https://cran.r-project.org/). Gene Set Enrichment Analysis (GSEA) is performed by using GSEA software 12. Hierarchical clustering and Principal Component Analysis were performed using iDEP. 93 13. For the gene expression data analysis of the sensitive and resistant cells, their gene count data that is produced by the Cancer Cell Line Encyclopedia (CCLE) was downloaded from the Depmap portal 14, and analyzed similarly to the analysis of BT549 cells treated with BDP-9066. The RNA-seq raw data is available in NCBI Sequence Read Archive (BioProject Accession: PRJNA1026994).

### Analysis of TCGA and METABRIC datasets, and breast cancer patient survival data

TCGA data were downloaded from UCSD Xena (http://xena.ucsc.edu/) and cBioPortal (https://www.cbioportal.org/). METABRIC data were downloaded from cBioportal. The data extraction of particular genes from the all gene count data, the statistical analyses, and graphs creation were performed using R's basic package and Tidyverse package. 22 previously reported genes were used as the gene cluster for calculating the YAP/TAZ target score 15. To calculate the Focal adhesion score, we used 408 genes confirmed to be expressed in breast cancer from the METABRIC dataset, among about 420 genes involved in focal adhesion from gene ontology data, which was obtained in the g: Profiler website 11. Breast cancer patient survival was analyzed by using Kaplan-Meier Plotter 16.

### Cell lines, plasmid, chemicals

SUM159 cells were obtained from Elabscience (Houston, TX, USA). The other cell lines were obtained from ATCC (American Type Culture Collection, USA). Cells were maintained in DMEM/F16 supplemented with 10% fetal bovine serum and 1% antibiotics. All cell lines used in this study were authenticated. Mutant YAP, shRNA against YAP, YAP-responsive luciferase plasmids were described previously 17. Non-targeting control, *CDC42BPA* and *CDC42BPB* smart pool siRNAs were purchased from Horizon Discovery (Waterbeach, UK).

### Transfection, lentiviral infection, luciferase assay

Lentivirus production and infection were performed as previously described 17. For luciferase assay, plasmid DNA transfection was conducted with use of Lipofectamine 2000 (ThermoFisher Scientific) according to the manufacturer's instructions. Luciferase assay was performed as described previously 18, 19. siRNAs were transfected with Lipofectamine RNAiMAX Transfection Reagent (ThermoFisher Scientific) according to the manufacturer's instructions.

### Cell viability assay (colony formation assay)

**Figure 1:**
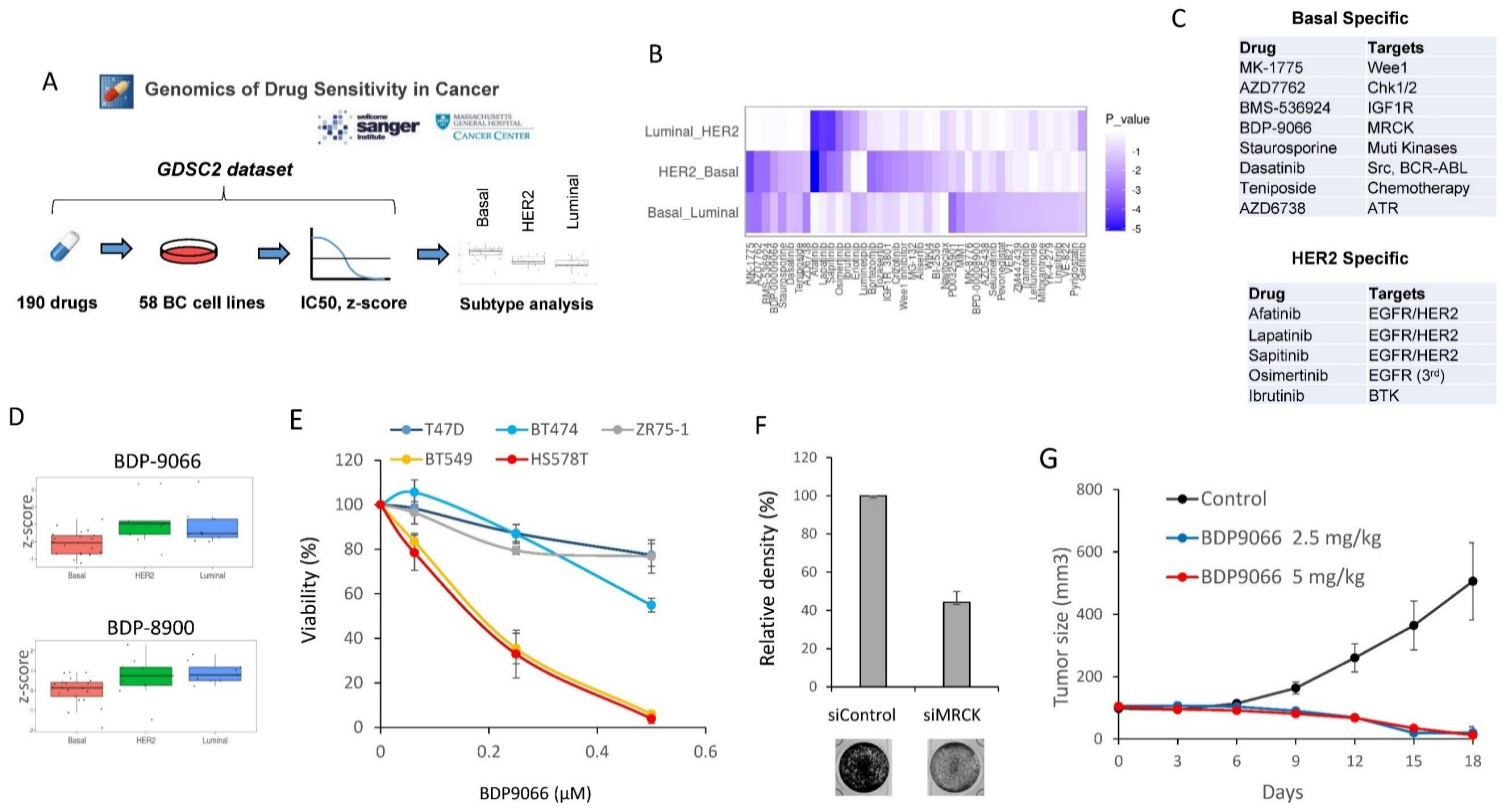
** MRCKi may serve as specific therapeutic agents for TNBC. (A)** The outline of the drug screening by analysis of the Genomics of Drug Sensitivity database. **(B)** The drugs that show significant differences between subtypes. The heat map shows the P-values for the IC50 differences of each drug between the two subtypes. The vertical axis represents the two subtypes compared and the horizontal axis represents the type of drug. **(C)** The list of Basal-specific or HER2-specific drugs identified by the screening. Drugs with significantly higher IC50s for both HER2 and Luminal compared to Basal are defined as Basal-specific drugs. Similarly, drugs with significantly higher IC50s for both Basal and Luminal compared to HER2 are defined as HER2-specific drugs. **(D)** The distribution of IC50 for each subtype of cells against BDP-9066 (upper) and BDP-8900 (lower) is shown in boxplots with jitter. P-values of between each subtype in BDP-9066 are following; HER2-Basal: 0.0052852, Basal-Luminal: 0.037525, and Luminal-HER2: 0.7883976. P-values of between each subtype in BDP-8900 are following; HER2-Basal: 0.0896867, Basal-Luminal: 0.0160972, and Luminal-HER2: 0.8032067. **(E)** The effect of BDP-9066 on various breast cancer cell lines was determined in a colony formation assay (mean ± sd, n=3). **(F)** *CDC42BPA* and *CDC42BPB* were knocked down in BT549 cells. The cells were then cultured for 5 days and stained with crystal violet. The relative density of cells was shown in the graph (n=3). **(G)** The effect of BDP-9066 on breast tumor growth was examined by a BT549 xenograft model (mean ± sd, n=10).

125-1000 cells were seeded in 24 plates in treated with various concentration of drugs for 10-14 days. The cells were fixed in 3.7% of formalin and stained with 5% crystal violet. For the experiment shown in Figure 1F, we knocked down both *CDC42BPA* and *CDC42BPB* genes and seeded the 10,000 cells in 12 well plates. To quantify the data, the crystal violet of the stained cells was extracted with 5% SDS solution and measure the absorbance at OD570, and the data was presented as percentage of untreated controls. All colony formation assay was performed in three-independent experiments, and the data was represented as mean ± standard deviation.

### Mouse xenograft models and toxicity study

2x 10^6^ of BT549 cells were injected into the mammary fat pads of female nude (Nu/Nu) mice of 6 weeks of age (from Lasco Biotechnology, Yi-Lan, Taiwan). When the tumor volume reached ~100 mm3, BDP-9066 dissolved in 20% PEG300-PBS, were injected subcutaneously to mice 2 times/daily for 18 days. Treatment was discontinued after 3 weeks because some mice in the control group had tumors exceeding 1000 mm3 in size. Tumors were measured at the indicated time points, and tumor volume was calculated by the formula: 1/2 × length × width^2^.

### Western blotting

Immunoblots were performed according to standard procedures 19. The antibodies used for this study are following; The antibodies against AKT (2920S), p-AKT (4060S), YAP (14074S), p-YAP (13008), TAZ (83669S), p-TAZ (59971S), p65 (6956S), p-p65 (3033S) are obtained from Cell Signaling Technology (Danvers, MA, USA). Anti-MRCKα (sc-374568) and anti-MRCKβ (sc-374597) antibodies were purchased from Santa Cruz Biotechnology (Dallas, TX, USA). The antibody for beta-actin (MA1-91399) was purchased from ThermoFisher Scientific. Anti-tubulin antibody (T5168) was purchased form Sigma-Aldrich (St. Louis, MO, USA). The chemiluminescent signals were detected by using iBright Imaging System (ThermoFisher Scientific). Signal intensity was measured using ImageJ software (https://imagej.nih.gov/ij/index.html). Ponceau S staining was used to confirm that the samples were equally loaded.

### Cell cycle analysis

Cells treated with/without BDP-9066 were trypsinized and washed. Cells were then fixed with 95% ethanol and their DNA was then stained with propidium iodide in the presence of RNase. The data was analyzed using the CytoFLEX Flow Cytometer (Beckman Coulter, Indianapolis, IN, USA).

### Quantitative PCR

BT549 cells were transfected with the indicated siRNA and cultured for 3 days. Total RNA was isolated with TRIZOL solution and cDNA was synthesized using PrimeScript™ RT reagent Kit (TaKaRa Bio, Shiga, Japan). qPCR was performed using ChamQ Universal SYBR qPCR Master Mix (Vazyme, Nanjing, China). The primers used for the experiments are following; CCN1-F: TGCCGCCTTGTGAAAGAAAC, CCN1-R: GCTGCATTTCTTGCCCTTTTTC, CCN2-F: CCGGGTTACCAATGACAACG, CCN2-R: TGCACTTTTTGCCCTTCTTAAT, Actin-F: GTCATTCCAAATATGAGATGCGT, Actin-R: GCTATCACCTCCCCTGTGTG.

### Statistics

All statistical analyses were performed using R. Statistical comparisons between two samples were performed using the Student's t-test, while one-way analysis of variance (ANOVA) with post-hoc Tukey HSD test was used for the comparison of multiple groups. A p-value of < 0.05 was considered significant.

## Results

### MRCKi act as specific agents against TNBC

To initially search for new drugs with specific activity against TNBC, we performed drug screening using a large publicly available database, *Drug Sensitivity Genomics in Cancer*, which contains analyzes of responses to over 500 different drugs in approximately 1,000 different cell lines 20. We extracted the data of 58 types of breast cancer cell lines from the dataset, and further classified the cells into three subtypes including Luminal, Basal, and HER2, based on the cell line data of the DepMap Portal 14. We then compared the z-score of IC50 data for each drug in each subtype (Fig. 1A). When one-way ANOVA was used to search for drugs with differences in each subtype, 42 types of drugs were identified (Fig. 1B). Furthermore, we defined those with significantly lower IC50 against Basal than both IC50 against HER2 and IC50 against Luminal as Basal (TNBC)-specific drugs. HER2-specific drugs were also identified in a similar manner. (Fig. 1C). All HER2 subtype-specific agents are RTK inhibitors, and all except one were HER2 inhibitors, confirming the success of this screening (Fig. 1C and Supplementary Fig. S1A). On the other hand, the drugs specific to Basal subtype include various drugs such as a chemotherapeutic agent, IGFR inhibitor, ATM inhibitor, and Aurora kinase inhibitor, most of which have previously been proven to be an effective drug against TNBC 21-23 (Fig. 1B and Supplementary Fig. S1B). Additionally, in the Basal subtype-specific drug list, we found BDP-9066, a myotonic dystrophic kinase-related Cdc42-binding kinase (MRCK) inhibitor that has never been reported as a drug against TNBC (Fig. 1C and 1D). The database also has another MRCKi, BDP-8900, which also tended to have a strong effect on Basal (Basal vs. Luminal: p=0.0160972 and Basal vs. HER2: p=0.0896867) (Fig. 1D). These results prompted us to further study MRCKi as a specific drug for targeting TNBC. Because BDP-9066 shows slightly better specificity for TNBC than BDP-8900, we first examined the effects of BDP-9066 on several breast cancer cell lines to validate the results of our drug screening with database analysis. Consistent with the screening data, TNBC cell lines BT549 and HS578T were highly sensitive to BDP-9066, whereas non-TNBC cell lines T47D, ZR75-1 and BT474 were more resistant to BDP-9066 than the TNBC cell lines (Fig. 1E). To further confirm that MRCK is indeed a target of TNBC, we knocked down the genes encoding two major MRCK isoforms, MRCKα and MRCKβ (*CDC42BPA* and *CDC42BPB*, respectively) and examined their effects on TNBC cell proliferation. In fact, when these genes were knocked down, the MRCK protein expression was significantly suppressed (Supplementary Fig. S1C) and cell proliferation was also suppressed (Fig. 1F). Furthermore, to investigate whether BDP-9066 can suppress TNBC tumor growth *in vivo*, we examined its effect using the BT549 Xenograft model. Consistently with the effects of BDP-9066 *in vitro*, both administration of BDP-9066 at 2.5 mg/kg and 5 mg/kg significantly inhibited tumor growth compared to the control group (Fig. 1G). When the mice treated with BDP-9066 were further maintained for 40 days after stopping drug administration, no tumors were detected in all except one mouse at each concentration group (90% cure rate, data not shown). Furthermore, no significant changes in body weight were observed during the drug administration, suggesting no significant toxicity at concentrations that could suppress TNBC growth *in vivo* (Supplementary Fig. S1D). These results indicated that BDP-9066, an MRCKi, may be an effective drug against TNBC.

### Pathways activated in MRCKi-sensitive cell lines

MRCK is a kinase that functions downstream of Cdc42 (Supplementary Fig. S2A). BDP-9066 is a highly specific inhibitor of MRCK and was first reported in 2018 as an effective drug against skin cancer 24. Furthermore, in the same year, BDP-9066 was shown to increase survival time in combination with radiation in a Glioblastoma xenograft model 25. In addition, BDP-9066 has been proven effective against ovarian cancer cells in 2020 26. ROCK, which functions downstream of Rho, belongs to the DMPK family along with MRCK, and these kinases share common substrates (Supplementary Fig. S2A) 10. Interestingly, in our screening data, GSK269962A, a specific inhibitor for ROCK, shows no specificity for TNBC (Supplementary Fig. S2B), suggesting that the Cdc42-MRCK pathway has a more dominant role for survival and proliferation of TNBC cells than the Rho-ROCK pathway.

**Figure 2:**
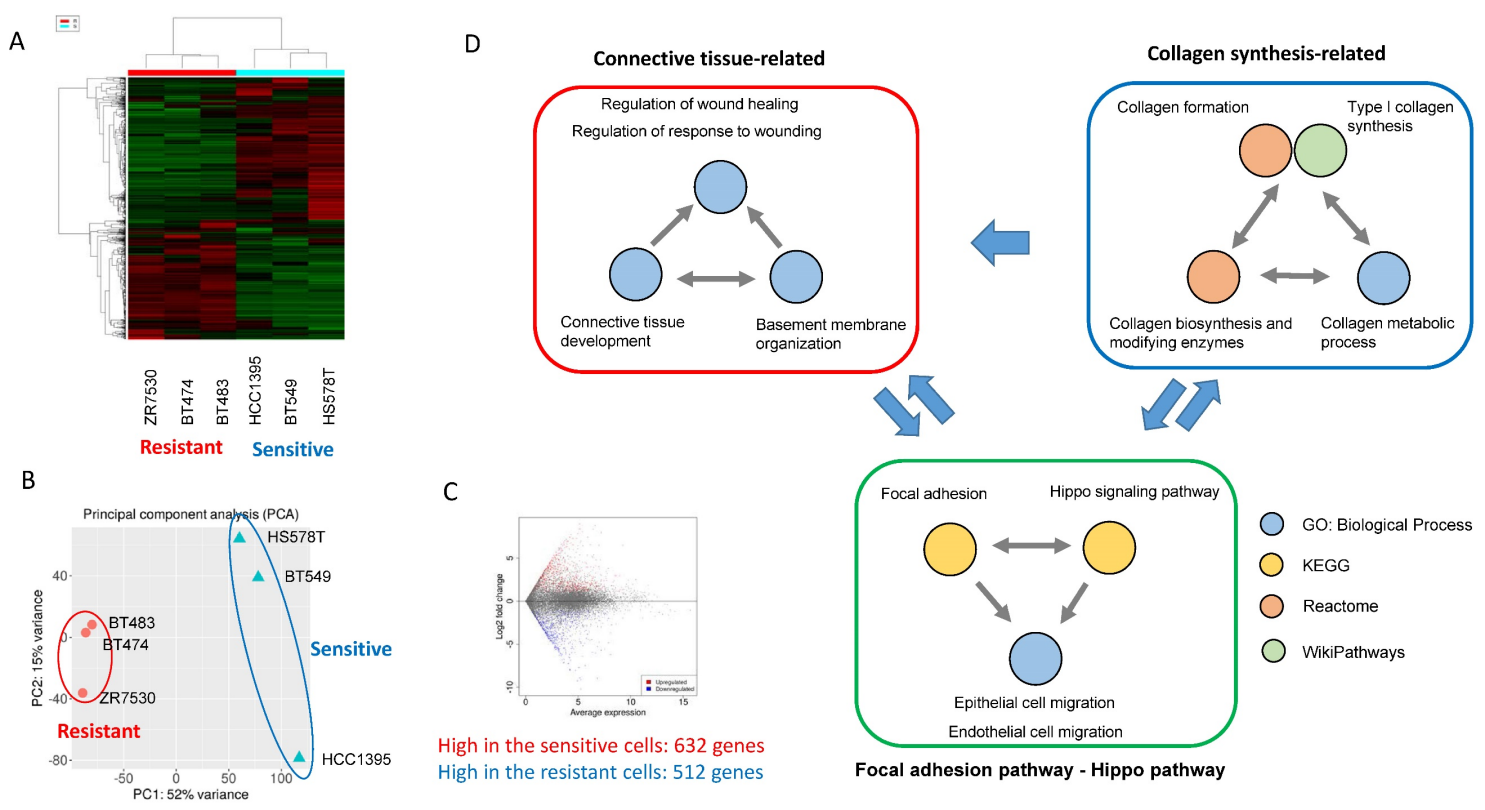
** Analysis of the mechanisms underlying the effects of BDP-9066 using gene expression profiles of sensitive and resistant cell lines. (A)** Hierarchical clustering by differentially expressed genes. The sensitive and resistant cell lines for BDP-9066 are fully classified. The sensitive cell lines include BT549, HCC1395, HS578T and the resistant ones include BT474, BT483, ZR7530. **(B)** Principal component analysis was performed with the gene expression profiles of the sensitive and resistant cell lines used in A. Similar to the result of A, the cells are divided into the sensitive and resistant cell lines. **(C)** MA plots representing differentially expressed genes between sensitive and resistant cell lines. **(D)** A summary of the results of pathway analysis performed using a group of genes that are specifically highly expressed in the sensitive cell lines. The results of pathway analysis are shown in Supplementary Fig. S2. Pathways found in Gene Ontology (Biological Process) and 3 pathway databases (KEGG, WikiPathways, Reactome) are shown in the same color, respectively. Also, the possible relationship between each group is indicated by arrows.

Next, to investigate the mechanism that explains the TNBC specificity of MRCKi, we selected three types of BDP-9066-sensitive (BT549, HS578T, and HCC1395) and resistant cells (ZR7530, BT474, and BT483) from the drug screening data performed in Fig. 1 and analyzed their gene expression profiles, which are obtained from the Cancer Cell Line Encyclopedia (CCLE) 27. First, we performed hierarchical clustering (Fig. 2A) and principal component analysis (Fig. 2B) to investigate whether they are grouped by sensitivity to BDP-9066. As a result, we were able to clearly distinguish between the resistant and sensitive cell lines, suggesting that differentially expressed genes in these cell lines are likely responsible for sensitivity to MRCKi. Therefore, to investigate which pathways are activated in cells sensitive to MRCKi, we extracted differentially expressed genes in sensitive and resistant cell lines (Fig. 2C) and performed pathway analysis. First, we analyzed genes highly expressed in the sensitive cell lines using WikiPathways, KEGG, and Reactome databases, and identified 15 dominantly activated pathways (Supplementary Fig. S2C).

In addition, Gene Ontology analysis revealed 230 Biological Process (BP) with a P value of 0.05 or less (The top 20 are shown in Supplementary Fig. S2D). Many of these pathways were found to be involved in the same function. Therefore, we extracted functionally common signals from these pathway analysis data and grouped them (Fig. 2D). First, we were able to group them into pathways involved in collagen synthesis and pathways involved in connective tissue formation (Fig. 2D). These two large functional groups are interrelated since collagen is necessary for the formation of connective tissue. More interestingly, Hippo pathway and focal adhesion were identified, both of which are known to be involved in Epithelial-mesenchymal transition (EMT) and cell migration 28, 29.

YAP and TAZ are transcriptional activators downstream of the Hippo pathway and oncogenic factors that induce multiple cellular responses such as cell proliferation, drug resistance, and metastasis in human solid tumor cells 30. YAP and TAZ are suppressed by phosphorylation by the MST-LATS pathway, which function as tumor suppressors (Supplementary Fig. S3A). MST/LATS is regulated by a variety of extracellular signals, including tight junction-mediated contact inhibition 31, mechanical cues 32 that regulate tissue regeneration and wound repair, focal adhesion, and GPCRs 28, 33 (Supplementary Fig. S3A).

In fact, YAP/TAZ promotes collagen synthesis, while collagen binds to integrins, activates signals from focal adhesions, and further activates downstream YAP/TAZ 34, 35. YAP/TAZ also promotes focal adhesions and EMT formation. Therefore, all these pathways are closely related to each other and breast cancer cells with active YAP/TAZ and focal adhesion pathways may be particularly sensitive to MRCKi.

We also analyzed genes that were highly expressed in resistant cell lines compared to sensitive cell lines. As a result, pathways specifically involved in tight junctions and epithelial cell polarity were identified (Supplementary Fig. S3B and S3C). Signals from tight junctions activate LATS and repress YAP/TAZ 31 (Supplementary Fig. S3A). Also, epithelial cells exhibit very weak cell migration activity. Thus, clearly opposite signals are activated in the resistant and sensitive cell lines. Taken together, the sensitive cell lines have the characteristics of mesenchymal cells in which YAP/TAZ and focal adhesion are activated, while the resistant cell lines have the characteristics of epithelial cells in which tight junctions are activated.

### Significance of YAP/TAZ in MRCKi sensitivity

**Figure 3:**
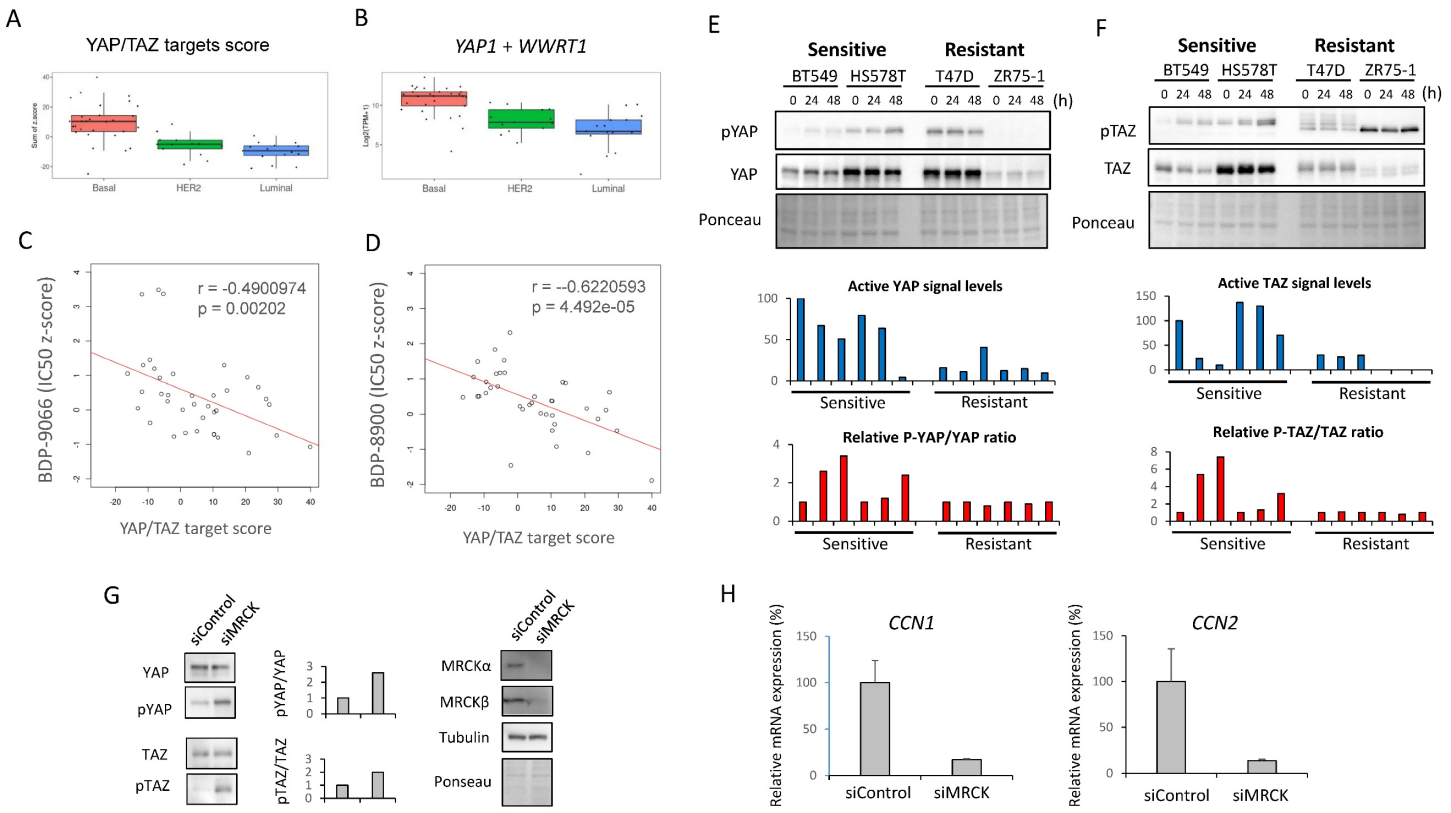
** Relationship between sensitivity to MRCKi and YAP/TAZ activity in breast cancer cell lines. (A)** The YAP/TAZ target scores in the breast cancer cell lines used for the screening in Fig. 1 is shown in the boxplot with jetters by subtype. Basal shows significantly higher values than HER2 and Luminal (P-value between each subtype; HER2 - Basal: 0.0001864, Luminal - Basal: 0.0000008, Luminal - HER2: 0.5061786). **(B)** The *YAP1 plus WWTR1* expression in the breast cancer cell lines used for the screening in Fig. 1 is shown in the boxplot with jetters by subtype. Basal shows significantly higher values than HER2 and Luminal (P-value between each subtype; HER2 - Basal: 0.0001864, Basal - Luminal: 0.0000008, Luminal - HER2: 0.5061786). **(C and D)** The relationship between the YAP/TAZ target score and susceptibility to MRCKi was shown in a scatter plot including a regression line. The YAP/TAZ target score is plotted on the X-axis and the IC50 against BDP-9066 **(C)** or BDP-8900 **(D)** on the Y-axis. There was a negative correlation between YAP/TAZ scores and IC50s for MRCKi. **(E and F)** The effect of BDP-9066 on YAP **(E)** and TAZ **(F)** phosphorylation and expression was examined by Western blotting. Signal intensities were obtained using ImageJ. The result obtained by subtracting the phosphorylated YAP/TAZ signal from the total YAP/TAZ signal is shown in the blue graph below as the activated YAP/TAZ signal. All values were calculated relative to the untreated BT549 as 100. Also, since the result of ZR75-1 was negative, it was set to 0 in the graph. The red graph shows the change in the ratio of the phosphorylation signal to the total signal. It was shown as a ratio to the untreated sample in each cell. **(G and H)** *CDC42BPA* and *CDC42BPB* were knocked down in BT549 cells and the expression of YAP/TAZ and phosphorylated YAP/TAZ expression were determined by Western blotting **(G)**. The relative ratios of p-YAP/YAP or p-TAZ/TAZ are shown in the middle graph. The expression of *CCN1* and *CCN2* in B549 cells were determined by quantitative PCR **(H)**. Relative expression to actin were shown (*CCN1*: p<0.01, *CCN2*: p=0.013).

Based on the above observations, we made the following hypotheses (Supplementary Fig. S4A); in certain TNBC cells, focal adhesion-activated MRCK is involved in the activation of YAP/TAZ, and activated YAP/TAZ further promotes collagen synthesis and focal adhesion formation; this feedforward loop is essential for the survival of such TNBC cells; moreover, MRCK and YAP/TAZ play a central role in this feedforward loop and inhibition of MRCK can inhibit such TNBC. Therefore, we next investigated whether YAP/TAZ actually functions downstream of MRCK in TNBC cells. First, in order to find an index of YAP/TAZ activity, we focused on YAP/TAZ target gene expression. A previous study identified 22 genes as core targets of YAP/TAZ from multiple-omics data, such as ChIP-sequencing and RNA-sequencing 15. Therefore, the sum of the Z-scores of the expression values of these 22 genes was used as the YAP/TAZ target score, and the YAP/TAZ target scores of the 58 types of breast cancer cells used in the screening of Fig. 1 were first examined (Fig. 3A). Also, the sum of the RNA counts for *YAP1* and *WWTR1* (YAP and TAZ genes, respectively) was calculated (Fig. 3B). The results showed that both YAP/TAZ activity and their mRNA expression were highest in the Basal subtype (Fig. 3A and 3B).

We also examined the expression of *CDC42BPA* and *CDC42BPB* (MRCKα and MRCKβ genes, respectively), but found no difference between any subtypes (Supplementary Fig. S4B). Next, we examined the correlation between the YAP/TAZ target score and IC50 to MRCKi in the breast cancer cell lines used in Fig. 1 (Fig. 3C and 3D). The results indicated that there was a significant negative correlation between them. In other words, cells with higher YAP/TAZ activity tend to be more sensitive to MRCKi. Furthermore, the expression and phosphorylation status of YAP/TAZ and the effect of BDP-9066 on them in the sensitive and resistant cell lines were examined by Western blotting. Phosphorylated YAP/TAZ are considered as inactive form. Therefore, when the level of activated YAP/TAZ signal was determined by subtracting the phosphorylated YAP/TAZ (p-YAP/p-TAZ) signal from the total YAP/TAZ signal, the level of activated YAP/TAZ signal tended to be higher in the sensitive cell lines (The blue graph in Fig. 3E and 3F). However, YAP/TAZ phosphorylation was strongly induced by BDP-9066 in the sensitive cell lines, but not in the resistant lines (The red graph in Fig. 3E and 3F). These results indicate that breast cancer cells with high YAP/TAZ activity are more sensitive to MRCKi and that such cells are particularly found in the TNBC subtype. Finally, to further verify the role of MRCK on YAP/TAZ, we knocked down *CDC42BPA* and *CDC42BPB* and examined their effects on YAP/TAZ phosphorylation. Consistent with BDP-9066 treatment, we observed that knockdown of MRCK increases the phosphorylation of YAP/TAZ (Fig. 3G). Moreover, the expression of YAP/TAZ targets, *CCN1* and *CCN2* expression was also downregulated by MRCK knockdown (Fig. 3H). Together, MRCK is the upstream regulators of YAP/TAZ in TNBC cells.

### YAP/TAZ is suppressed by BDP-9066, and constitutively active YAP reverse the effect of BDP-9066

**Figure 4:**
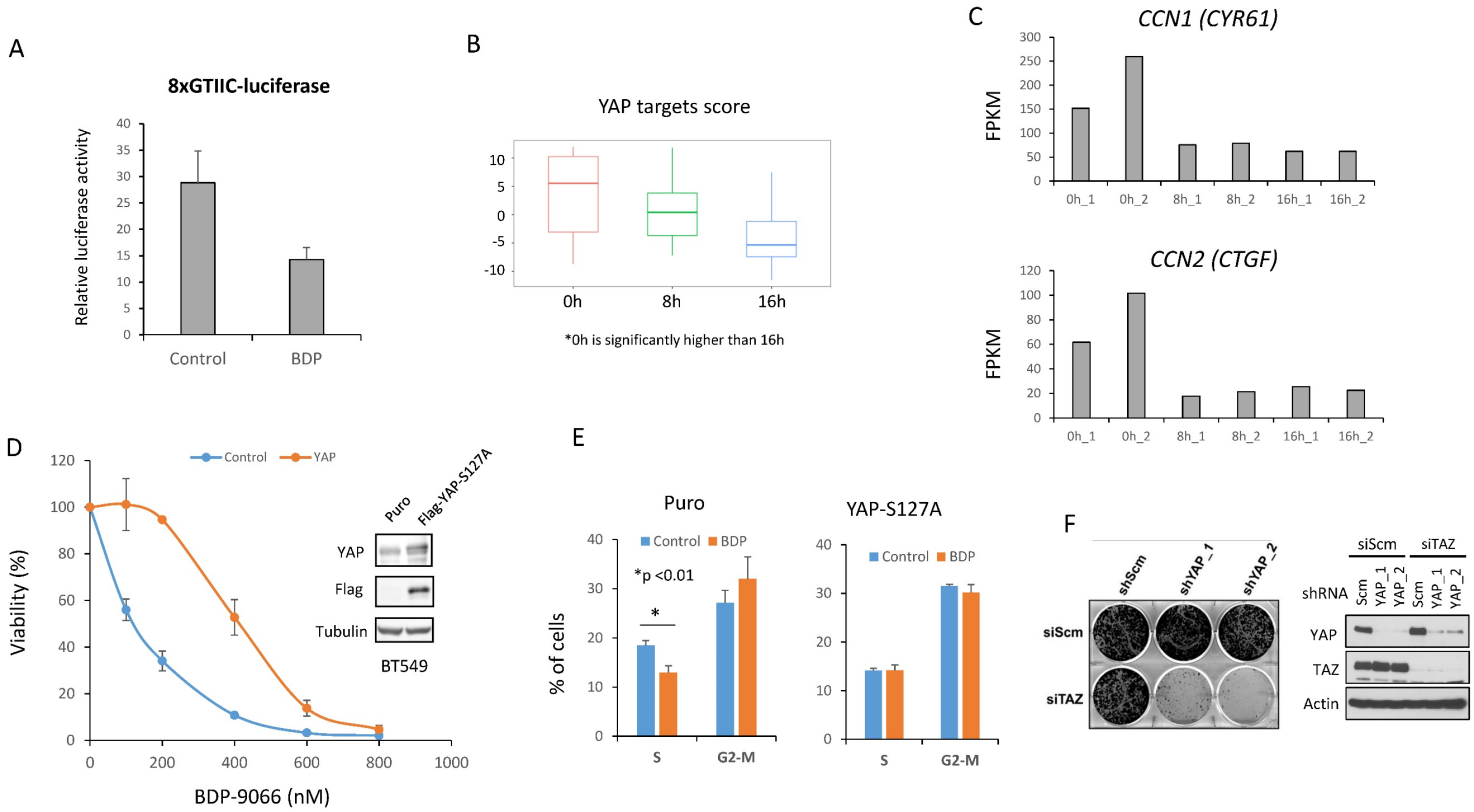
** Suppression of YAP/TAZ is necessary for the effect of BDP-9066. (A)** BT549 cells were transiently transfected with YAP/TAZ-controlled luciferase plasmids and treated/untreated with BDP-9066 for 8 h before harvesting samples and measuring relative luciferase activity. **(B)** After treating BT549 cells with 0.5 mM BDP-9066 for 8 or 16 hours, total RNA was harvested and used for RNA sequencing. We then calculated the YAP/TAZ target score for each time. **(C)** The RNA count data of *CCN1* and *CCN2* were extracted from the RNA sequencing data used in B. The data shows the results of two independent samples. **(D)** Colony formation assay was performed to test the sensitivity to BDP-9066 in constitutively active YAP-expressing BT549 cells and control BT549 cells. Active YAP significantly reduces sensitivity to BDP-9066. **(E)** The effect of BDP-9066 on the cell cycle was examined in YAP-expressing BT549 cells and control BT549 cells. **(F)** After knocking down YAP and TAZ in 231 cells with two types of shRNA and siRNA, respectively, 4000 cells each were seeded in a 6-well plate and cultured for 6 days. Knockdown efficiency was confirmed by Western blotting (right panel).

BDP-9066 treatment increased YAP/TAZ phosphorylation in the sensitive cell lines (Fig. 3E, F), suggesting that BDP-9066 treatment suppressed YAP/TAZ activity. Therefore, to investigate whether BDP-9066 actually downregulates YAP/TAZ activity, we examined the effect of BDP-9066 on YAP/TAZ activity using a luciferase plasmid whose transcriptional activity is regulated by YAP/TAZ. Consistent with the western blotting results, we observed that BDP-9066 reduced the activity of YAP/TAZ (Fig. 4A). Next, BT549 cells were treated with BDP-9088 for 8 hours and 16 hours, followed by RNA sequencing to examine changes in the YAP/TAZ target scores. Again, it was confirmed that BDP-9088 treatment significantly decreased the YAP/TAZ target score (Fig. 4B). In particular, the transcription of *CCN1* and *CCN2* (genes encoding Cysteine-rich angiogenic inducer 61 [CYR61] and Connective tissue growth factor [CTGF], respectively), which are the most important targets of YAP/TAZ, were most strongly suppressed (Fig. 4C). Next, to investigate whether the inhibition of YAP/TAZ by BDP-9066 is indeed responsible for the inhibition of cell proliferation by BDP-9066, we expressed a constitutively active YAP-S127A mutant in BT549 cells and investigated the effect of BDP-9066 by colony formation assay and cell cycle analysis. The results showed that active YAP attenuated the cell growth inhibitory effect of BDP-9066 (Fig. 4D) and also suppressed the cell cycle inhibitory effect of BDP-9066 (Fig. 4E). Furthermore, the importance of YAP/TAZ in TNBC cells was verified by the marked suppression of cell proliferation when both were simultaneously knocked down in TNBC cells (Fig 4F). These findings suggest that YAP/TAZ is an essential factor for TNBC that acts downstream of MRCK, and that suppression of YAP/TAZ is necessary for the effects of MRCKi.

### The signaling of focal adhesion and YAP/TAZ are critical for the Claudin-low subtype

**Figure 5:**
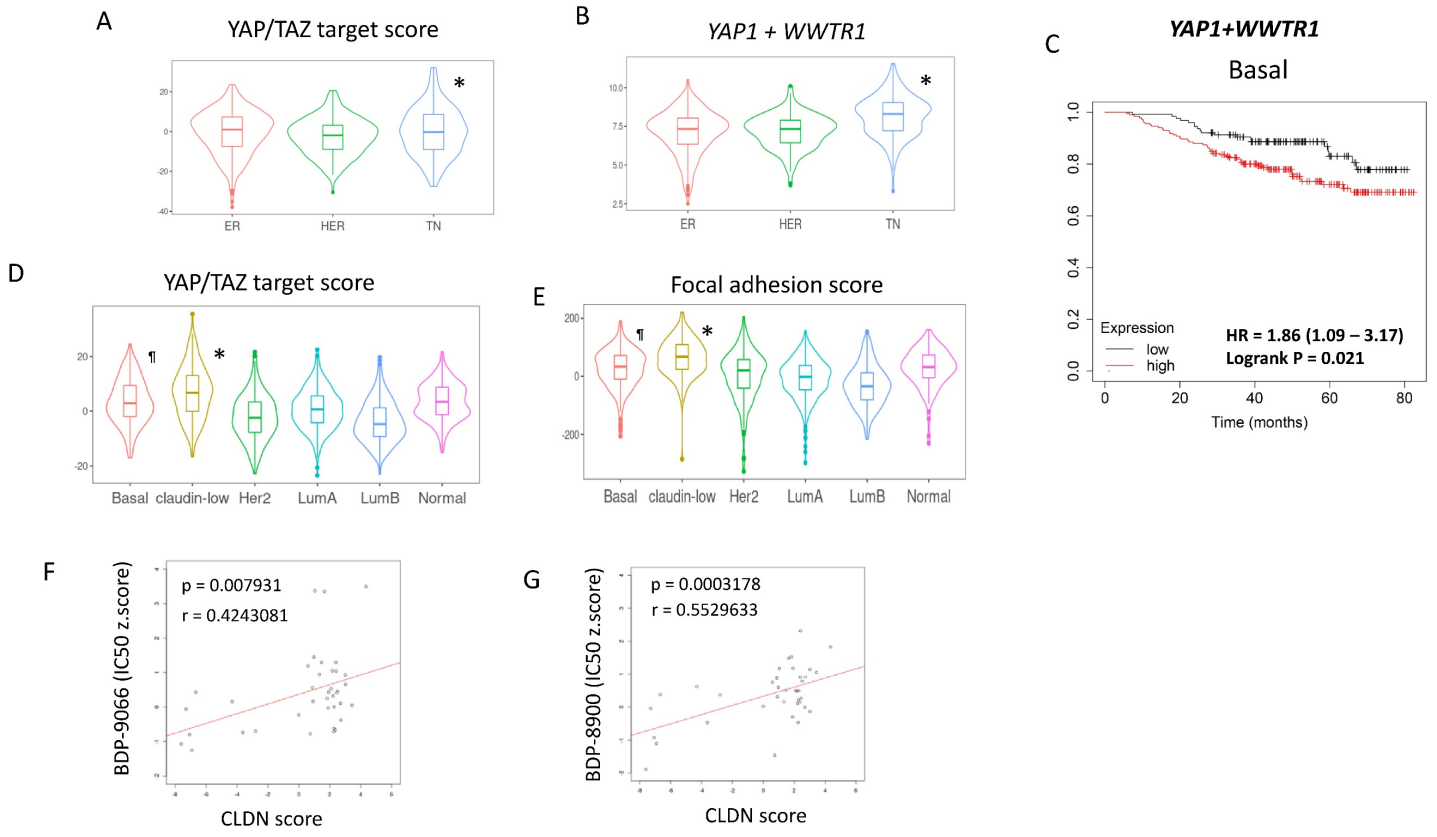
** Focal adhesion - MRCK - YAP/TAZ is particularly important for the Claudin-low subtype. (A and B)** Using TCGA breast cancer gene expression data, we examined the YAP/TAZ target scores and sum of *YAP1* and *WWTR1* in ER+ (ER), ER-/HER2+ (HER), and TNBC (TN) breast cancers. *TN shows the significantly higher YAP/TAZ target score as well as *YAP1*+*WWTR1* expression than other subtypes. **(C)** Breast cancer patients with Basal subtype were divided into two groups based on the high and low total *YAP1*/*WWTR1* expression levels, and their survival curves were compared (Logrank P=0.021). **(D)** We calculated the YAP/TAZ target scores for six intrinsic subtypes using the METABRIC dataset. *Claudin-low is significantly higher than any other subtype, and ^¶^Basal is significantly higher than HER2, Luminal A (LumA) and Luminal B (LumB) subtypes. **(E)** We calculated the Focal adhesion scores for six intrinsic subtypes using the METABRIC dataset. *Claudin-low is significantly higher than any other subtype, and ^¶^Basal is significantly higher than HER2, Luminal A (LumA), Luminal B (LumB) and Normal subtypes. **(F and G)** The relationship between the CLDN score and susceptibility to MRCKi in breast cancer cell lines is shown in scatterplots with regression lines. The CLDN scores are plotted on the X-axis and IC50 against BDP-9066 **(F)** or BDP-8900 **(G)** are plotted on the Y-axis. There is a positive correlation between the CLDN score and IC50 of MRCKi.

Next, we examined the importance of YAP/TAZ in TNBC in breast cancer clinical samples. First, the gene expression data of breast cancer from The Cancer Genome Atlas (TCGA) was classified into ER and/or PR+/HER2-, HER2+, TNBC by immunohistochemical staining (IHC) data. Then, the YAP/TAZ target score (Fig. 5A) and the sum of *YAP1* and *WWTR1* expression (Fig. 5B) were analyzed in each immunohistochemical subtype. The results showed significantly higher YAP/TAZ activity and gene expression in TNBC (Fig. 5A and 5B), similar to cell line data (Fig. 3A and 3B). Furthermore, we investigated the relationship between the sum of *YAP1* and *WWTR1* expression and survival in breast cancer patients with different intrinsic subtypes. While these gene expressions were associated with shorter survival in patients with Basal subtype and HER2 subtype, but not Luminal subtype (Fig. 5C, Supplementary Fig. S5A-5C).

Next, we performed a similar analysis using The Molecular Taxonomy of Breast Cancer International Consortium (METABRIC) dataset, which has over 2000 breast cancer gene mutations, gene expression and phenotype information 36. The METABRIC dataset is classified by Perou's intrinsic subtypes, including Claudin-low and normal subtypes 3. Therefore, the YAP/TAZ target scores were examined for six subtypes of breast cancer: Luminal A, Luminal B, HER2-enriched, Basal, Claudin-low, and Normal (Fig. 5D). At the same time, the sum of the z-scores of the expression of 408 genes involved in focal adhesion was also examined using the same dataset and we used it as the Focal adhesion score (Fig 5E). Both the YAP/TAZ target score and focal adhesion score were found to be highest in the Claudin-low subtype, followed by the Basal subtype (Fig. 5D and 5E). This is consistent with the classification of BT549 and HS578T, which are most susceptible to BDP-9066, into the Claudin-low subtype 37. The Claudin-low subtype is characterized by decreased expression of four genes, Claudin-3, 4, 7 and E-cadherin 38. Therefore, we define the sum of these expressions as the CLDN score, and the correlation between the IC50 for MRCKi and the CLDN score in breast cancer cells was investigated. The results showed that cells with lower CLDN scores have lower IC50s, indicating that lower Claudin expression is associated with greater sensitivity to MRCKi (Fig. 5F and 5G). Together, these findings suggest that the focal adhesion > MRCK > YAP/TAZ signaling loop plays an important role in TNBC, especially in the Claudin-low subtype, and MRCKi may be particularly effective drugs against the Claudin-low subtype breast cancer.

### MRCKi downregulate NF-κB activity in the sensitive cells

**Figure 6:**
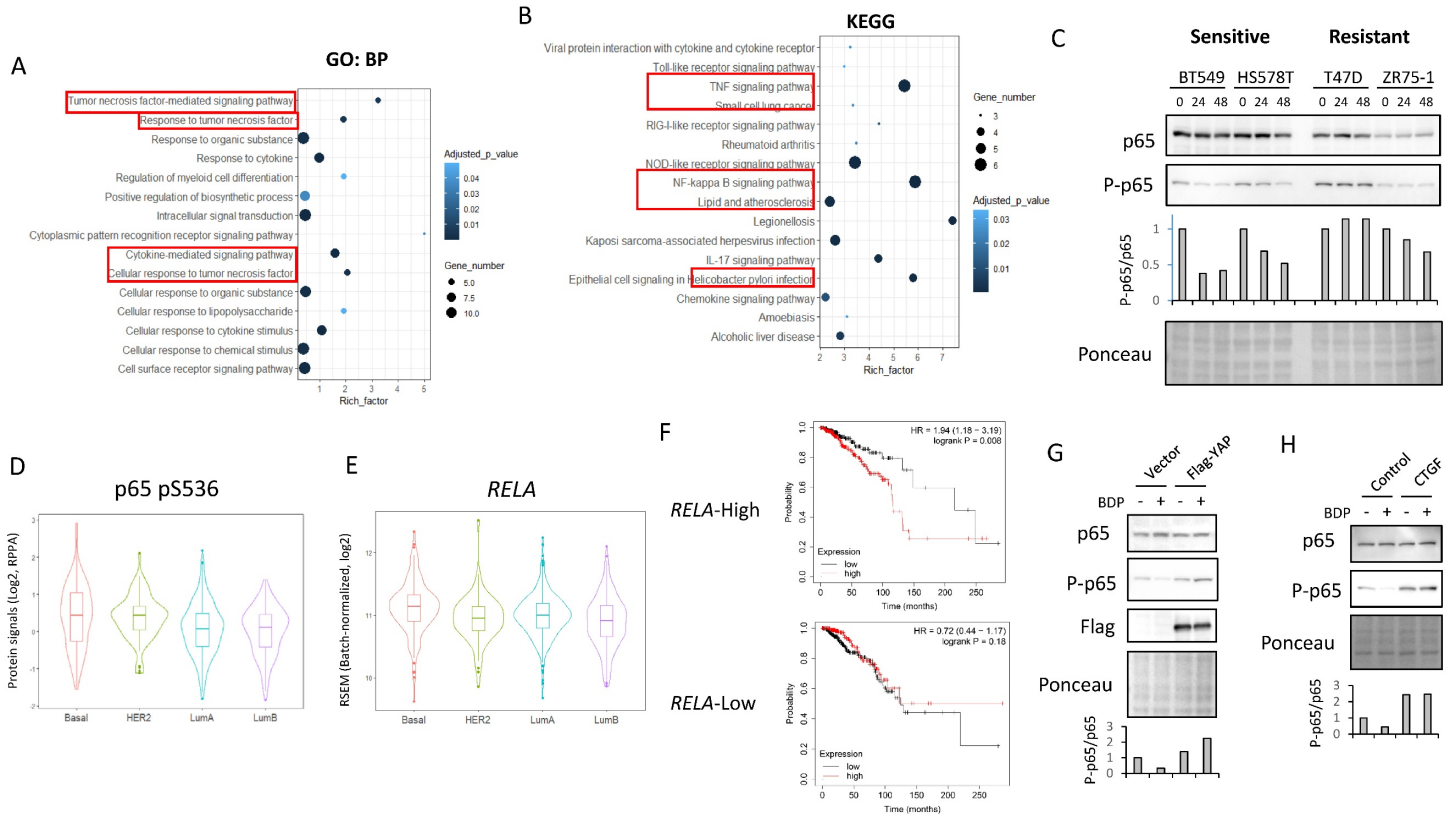
** NF-kB activity is suppressed by BDP-9066 via inhibition of YAP/TAZ. (A and B)** Pathway analysis was performed using genes that were downregulated in BT549 cells treated with BDP-9066. The results using Gene Ontology (Biological Process) **(A)** and KEGG **(B)** database are shown, respectively. **(C)** Western blotting was performed to analyze the expression levels of total p65 and phosphorylated p65 (pS536) after treatment with BDP-9066 in the sensitive and resistant cell lines. **(D)** The TCGA RPPA data was analyzed to determine p65 phosphorylation levels in different subtypes of breast cancer. The P value for each pair; P= HER2-Basal: 0.9804435, LumA-Basal: 0.0000022, LumB-Basal: 0.0000075, LumA-HER2: 0.0032619, LumB-HER2: 0.0024520, LumB-LumA: 0.9342813. **(E)** The TCGA RNA sequencing data was analyzed to determine *RELA* expression levels in different subtypes of breast cancer. The P value for each pair; HER2-Basal: 0.0019109, LumA-Basal: 0.0003119, LumB-Basal: 0.0000000, LumA-HER2: 0.6992430, LumB-HER2: 0.7464300, LumB-LumA: 0.0086262. **(F)** In the TCGA dataset, breast cancer patients were divided into a group with high *RELA* expression and a group with low *RELA* expression in their tumors, and the relationship between *YAP1* expression level and overall survival was investigated in each group. *YAP1* expression was associated with shorter survival in *RELA*-high group (p=0.008), but not *RELA*-low groups (p=0.18). **(G)** The effect of BDP-9066 on p65 phosphorylation in BT549 cells expressed constitutively activated YAP and the vector control cells was examined by Western blotting. **(H)** The effect of BDP-9066 on p65 phosphorylation in CTGF-treated or untreated BT549 cells was examined by Western blotting.

Next, to investigate whether MRCKi affect survival signals other than YAP/TAZ, we analyzed the RNA-sequencing data using samples from BT549 cells treated with BDP-9066. In particular, the pathways suppressed by MRCKi were determined by analyzing genes that were downregulated by BDP-9066. First, we performed hierarchical clustering and principal component analysis, and found that gene expression changed in a time-dependent manner (Supplementary Fig. S6A and S6B). Next, Genes significantly decreased at 8 hours and 16 hours compared to the control was extracted (Supplementary Fig. S6C and S6D). Then, we found that there are 20 genes whose expression level is decreased both at 8 hours and 16 hours compared to the control (Supplementary Fig. S6E). Pathway analysis using these genes identified NF-κB-related signals such as TNF, Toll-like receptor, IL-17, and NOD-like receptor (Fig. 6A and 6B). NF-κB is known to be involved in cancer cell survival, EMT, and drug resistance 39. Therefore, in order to investigate whether NF-κB is indeed suppressed by BDP-9066, we treated the sensitive or resistant cells with BDP-9066, and then analyzed p65 phosphorylation, which is an indicator of NF-κB activation, by Western blot. The results showed that BDP-9066 suppresses NF-κB activation particularly in sensitive cells (Fig. 6C). We also used the TCGA reversed-phase protein array (RPPA) data to investigate the phosphorylation status of p65 in four intrinsic subtypes of breast cancer, Luminal A, Luminal B, HER2 and Basal. As a result, we found that the Basal subtype had significantly higher phosphorylation of p65 than the Luminal subtypes (Fig. 6D). When we also examined the expression of *RELA* (the gene that encodes p65) in these subtypes, we found that it was significantly higher in the Basal type than in the other subtypes (Fig. 6E).

Furthermore, using the gene expression profiles of the resistant and sensitive cell lines used in Figure 2, we performed a gene set enrichment analysis (GSEA) and found that the TNFα - NF-κB pathway gene set was enriched in the sensitive cell lines (Supplementary Fig. S6F). Interestingly, in the group with high *RELA* expression, patients with cancer with high *YAP1* expression had significantly shorter survival times, but not in the group with low *RELA* expression (Fig. 6F). This suggests that the NF-κB pathway is important for the oncogenic function of YAP in breast cancer. Therefore, to investigate the possibility that YAP acts upstream of NF-κB, the effect of BDP-9066 on NF-κB was examined in cells expressing constitutively activated YAP. We found that constitutively active YAP suppresses the inhibitory effect of BDP-9066 on NF-κB phosphorylation (Fig. 6G). A previous study has shown that CTGF, the most important target of YAP/TAZ, can activate NF-κB 40. Therefore, to investigate whether CTGF is involved in YAP/TAZ-mediated NF-κB activation in TNBC, we examined the effect of BDP-9066 on NF-κB phosphorylation in the presence or absence of exogenous CTGF. In fact, we found that inhibition of NF-κB by BDP-9066 can be rescued by exogenous CTGF (Fig. 6H). Taken together, these findings suggest that NF-κB plays an important role in TNBC, and that NF-κB is also involved in the anti-TNBC action of BDP-9066. Moreover, YAP/TAZ is, at least in part, involved in NF-KB activation through induction of CTGF in TNBC (Supplementary Fig. S6G).

### Inhibition of PI3K-AKT pathway can potentiate the effects of BDP-9066 in PIK3CA mutant TNBC

**Figure 7:**
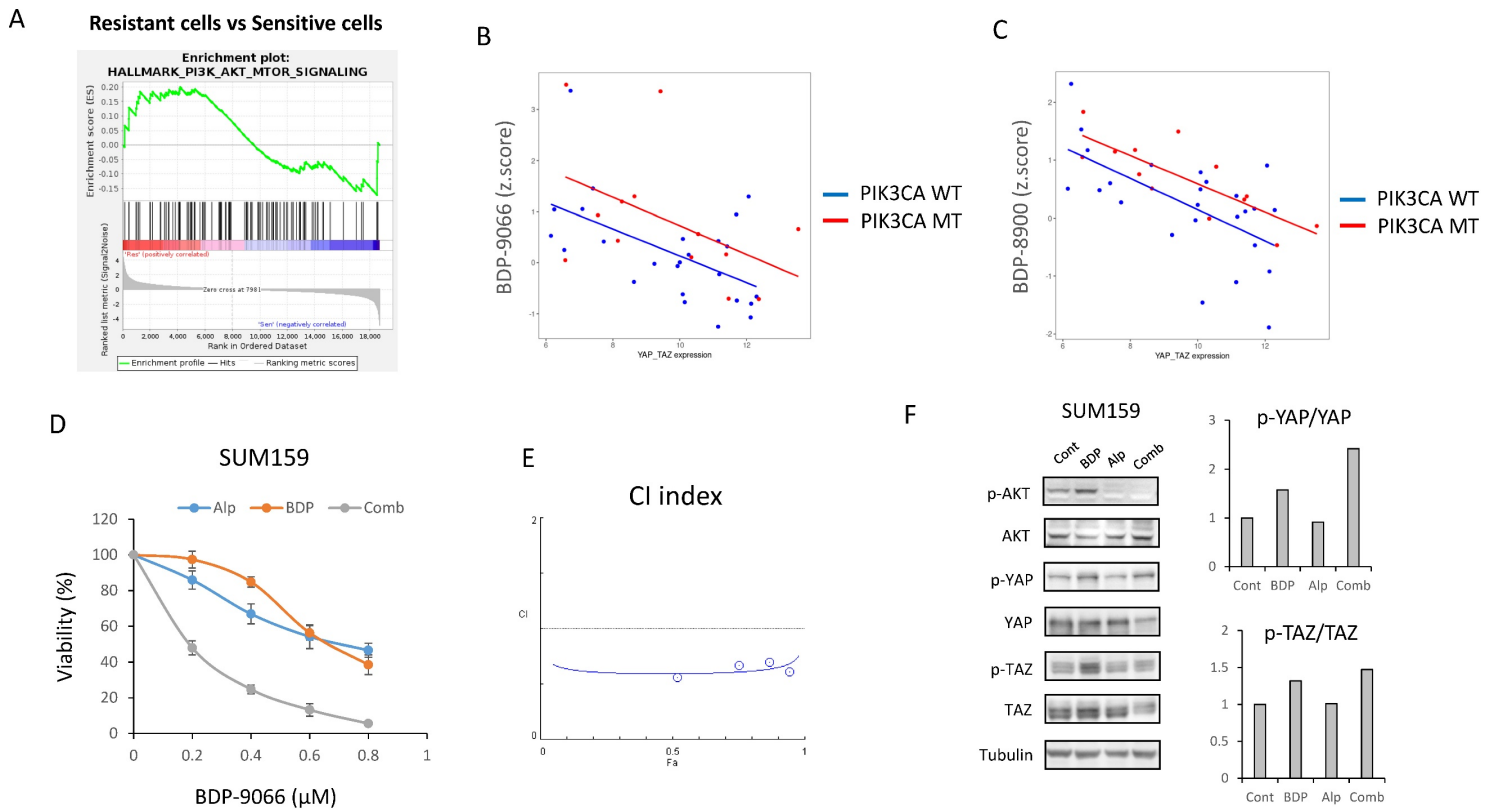
** Inhibition of PI3K-AKT pathway can potentiate the effects of BDP-9066 in PIK3CA mutant TNBC. (A)** Gene Set Enrichment Analysis with the hallmark gene sets was performed using the gene expression profiles of the resistant and sensitive cell lines used in Fig 2. Total 11 gene sets are upregulated in the resistant cells. HALLMARK_PI3K_AKT_MTOR_SIGNALING is in the list of enriched in the resistant phenotype. Normalized Enrichment score is 1.081 and Nominal p-value is 0.093. **(B and C)** The relationship between the *YAP1/WWTR1* expression and susceptibility to MRCKi in breast cancer cell lines are shown in scatterplots with regression lines. *PIK3CA* mutant cells are indicated by red dots and *PIK3CA* wild-type ones by blue dots, and the regression lines for each are also shown in the same color. *YAP1*/*WWTR1* expression on the X-axis and IC50 against BDP-9066 **(B)** or BDP-8900 **(C)** on the Y-axis. **(D)** The effects of BDP-9066, alpelisib, and their combination on viability of SUM159 cells were determined by colony formation assay. **(E)** The combination index was determined by the Chou-Talalay method. **(F)** The effects of BDP-9066, alpelisib, and their combination on YAP/TAZ phosphorylation in SUM159 cells were examined by Western blotting. The relative ratios of p-YAP/YAP or p-TAZ/TAZ are shown in the right graph.

The above results suggest that MRCKi could be effective drugs for TNBC, especially for Claudin-low subtype breast cancer. Next, to investigate potential agents that might enhance the efficacy of BDP-9066, we focused on signaling pathways specifically activated in cell lines resistant to BDP-9066. Then, we performed GSEA using the genes that are highly expressed in the resistant cell lines used in Fig. 2, and identified that the activation of the PI3-AKT kinase pathway was higher in the resistant cell lines than in the sensitive cell lines. Indeed, the PI3K-AKT pathway is known to regulate YAP/TAZ through various mechanisms (Supplementary Fig S7A). In the breast cancer cell lines used in Fig. 1, there is a negative correlation between *YAP1*/*WWTR1* expression and sensitivity to MRCKi. Then, we calculated regression lines only in cells with wild-type or mutants of the *PIK3CA* gene (PI3 kinase p110α subunit) and compared them to examine the effect of *PIK3CA* mutants on MRCKi sensitivity. As a result, *PIK3CA* mutant cells tended to be less sensitive to MRCKi regardless of *YAP1*/*WWTR1* expression levels. (Fig. 7B and 7C). This was not observed when the same analysis was performed with cells harboring mutant or wild type *TP53* gene (Supplementary Fig S7B and S7C). These results suggest that the PI3K pathway suppresses the effects of MRCKi, and PI3K inhibitors may potentiate the effects of MRCKi. Alpelisib, a PI3Kα inhibitor, is currently used as a therapeutic drug for ER-positive, HER2-negative, *PIK3CA*-mutated breast cancer 41. We therefore investigated the effect of different concentrations of the combination of BDP-9066 and alpelisib on SUM159 cells, a Claudin-low subtype harboring the PIK3CA mutant (Fig. 7D). Interestingly, SUM159 cells were relatively resistant to both drugs, but synergistic effects were seen with the combination (Fig. 7D and 7E). Furthermore, when the effects of these drug combinations on YAP/TAZ phosphorylation and expression were examined by Western blotting, it was found that the phosphorylation induction by BDP-9066 was further enhanced and their total levels were reduced by the addition of alpelisib (Fig. 7F). These results suggest that PI3K inhibitors can enhance the effects of MRCKi, and may be particularly effective against PIK3CA mutant TNBC.

## Discussion

In this study, we demonstrated that MRCKi are potential therapeutic agents for TNBC. Interestingly, although ROCK and MRCK phosphorylate the same substrates 10, our screening indicated that MRCKi show specificity for TNBC, whereas a ROCK inhibitor does not (Fig. 1D and Supplementary Fig. S2B). MRCK and ROCK generally function in different locations within the cell, and the importance of MRCK and ROCK varies by cell type 10. Thus, for example, ROCK or MRCK may be more important than the other in some cell types, while MRCK and ROCK may function equally important in some cell types.

In TNBC, especially the Claudin-low subtype, MRCK may function more significantly than ROCK, and MRCKi might be more effective against this subtype. If this hypothesis is correct, MRCKi may be ideal drugs with a wide therapeutic window against the Claudin-low subtype because ROCK functions to compensate for MRCK suppression in normal cells. In particular, BDP-9066, a highly selective inhibitor of MRCK, could be a very effective drug 24. MRCKi have so far only been tested in preclinical studies and have not reached clinical trials. In contrast, ROCK inhibitors are approved for non-cancer diseases such as ocular hypertension and open-angle glaucoma 42. AT13148, an inhibitor of ROCK and AKT, has also been tested in a Phase-I clinical trial against solid tumors but has not progressed to Phase-II due to a narrow therapeutic index and an inadequate pharmacokinetic profile 42.

Previous studies have shown that signals from GPCRs regulate YPA/TAZ via RhoA/ROCK 28. In particular, inhibition of LATS via actin polymerization is one of the important mechanisms for YPA/TAZ activation 32. Although MRCK plays an important role in actin polymerization similar to ROCK, there has been no report on YAP/TAZ regulation by MRCK. In this study, we demonstrated for the first time that MRCK plays an important role in regulating YAP/TAZ in TNBC cells.

Interestingly, we found that the NF-κB pathway was suppressed by inhibiting MRCK. NF-κB is a critical survival pathway for cancer cells, and this signal is also known to be involved in EMT and drug resistance 39, 43. Therefore, this would be one of the important mechanisms of anticancer activity by MRCKi (Supplementary Fig. S6G). In fact, various drugs that can suppress NF-κB have been studied and developed and are known to be effective against breast cancer cells 44. However, since NF-κB is involved in a variety of cellular functions, it is important to consider their toxicities when using NF-κB inhibitors. MRCKi would suppress YAP/TAZ-mediated NF-κB activation in TNBC and therefore may be less toxic and more effective than other NF-κB inhibitors in TNBC. Further research is needed to investigate this possibility. Furthermore, immune checkpoint inhibitors have been widely used in recent years, and it has been suggested that both the NF-κB and YAP/TAZ pathway is involved in their resistance 45, 46. Therefore, MRCKi may also increase the effectiveness of immune checkpoint inhibitors.

In addition, several specific inhibitors against YAP/TAZ are currently being developed 47, and they may also be effective drugs against TNBC, especially the Claudin-low subtype. However, YAP/TAZ is essential for the survival and proliferation of various cell types, suggesting that potent YAP/TAZ inhibitors may be highly toxic. On the other hand, given that the Focal adhesion-MRCK signal is likely dominant in the regulation of YAP/TAZ in Claudin-low subtype breast cancer, MRCKi may be preferred in this type of cancer.

Finally, we showed that alpelisib, a PI3 kinase inhibitor, enhances the effect of BDP-9066 and that *PIK3CA* mutations could be used as the biomarker for this combination. It has also been shown that YAP/TAZ inhibitors and AKT inhibitors actually exhibit a synergistic effect 47. Therefore, it is necessary to further investigate the combination of various PI3 kinase pathway inhibitors and BDP-9066. In particular, it is important to examine the synergistic effect of PI3 kinase inhibitors and MRCKi in various TNBC xenograft models. Moreover, the PI3 kinase and NF-κB pathways have been known to crosstalk through various mechanisms 48. As mentioned above, NF-κB is an important molecule downstream of the MRCK-YAP/TAZ pathway. Therefore, the PI3 kinase pathway may function as a bypass for NF-κB activation, and suppressing both PI3 kinase and MRCK may be effective in this regard as well.

## Supplementary Material

Supplementary figures.

## Abbreviations

CTGF: Connective tissue growth factor
EMT: epithelial-mesenchymal transition
FDA: Food and drug administration
GPCR: G-protein-coupled receptors
GSEA: Gene set enrichment analysis
HER2: human epidermal growth factor receptor 2
LIMK: LIM domain kinase
MCL2: myosin light chain 2
METABRIC: Molecular Taxonomy of Breast Cancer International Consortium
MRCK: myotonic dystrophy kinase-related Cdc42-binding kinase
MRCKi: MRCK inhibitor
MYPT1: myosin phosphatase target subunit-1
NF-κB: Nuclear factor kappa B
PI3: Phosphoinositide 3
ROCK: Rho-associated protein kinase
RTK: receptor tyrosine kinase
TAZ: the transcriptional coactivator with PDZ-binding motif
TCGA: The Cancer Genome Atlas
TNBC: Triple-negative breast cancer
YAP: Yes-associated protein
