# Supplementary material for: MRCK as a Potential Target for Claudin-Low Subtype of Breast Cancer
Source: Int J Biol Sci. 2024 Jan 1;20(1):1–14. doi: 10.7150/ijbs.88285 (PMC10750295; doi:10.7150/ijbs.88285)
Supplement: Supplementary file 1 — Supplementary figures. [file ijbsv20p0001s1.pdf]

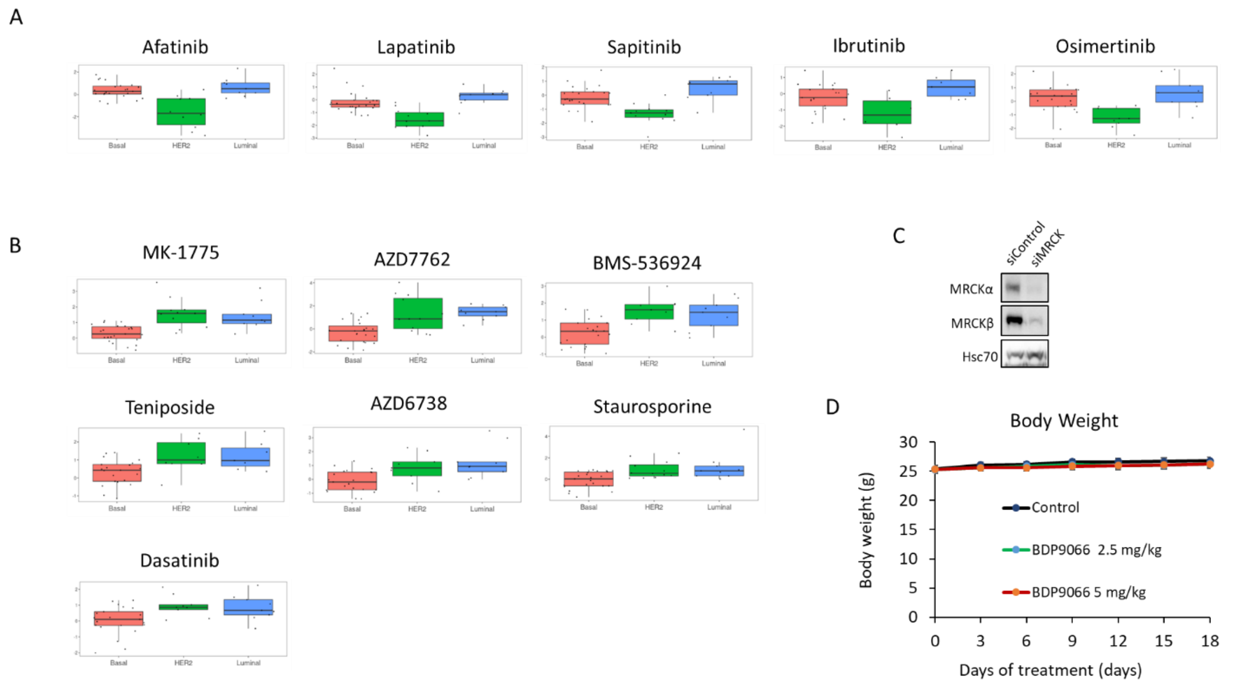

Supplementary Figure S1

(A) Boxplot with jetter showing IC50 of drugs specific for the HER2 subtype in Fig. 1C, excluding BDP-9066. **(B)** Boxplot with jetter showing IC50 of drugs specific for the Basal subtype in Fig. 1C. **(C)** The expression of MRCKα, MRCKβ and HSC70 in BT549 cells used in Fig. 1F. **(D)** Body weight during administration of BDP-9066 in mice used in Fig. 1G.

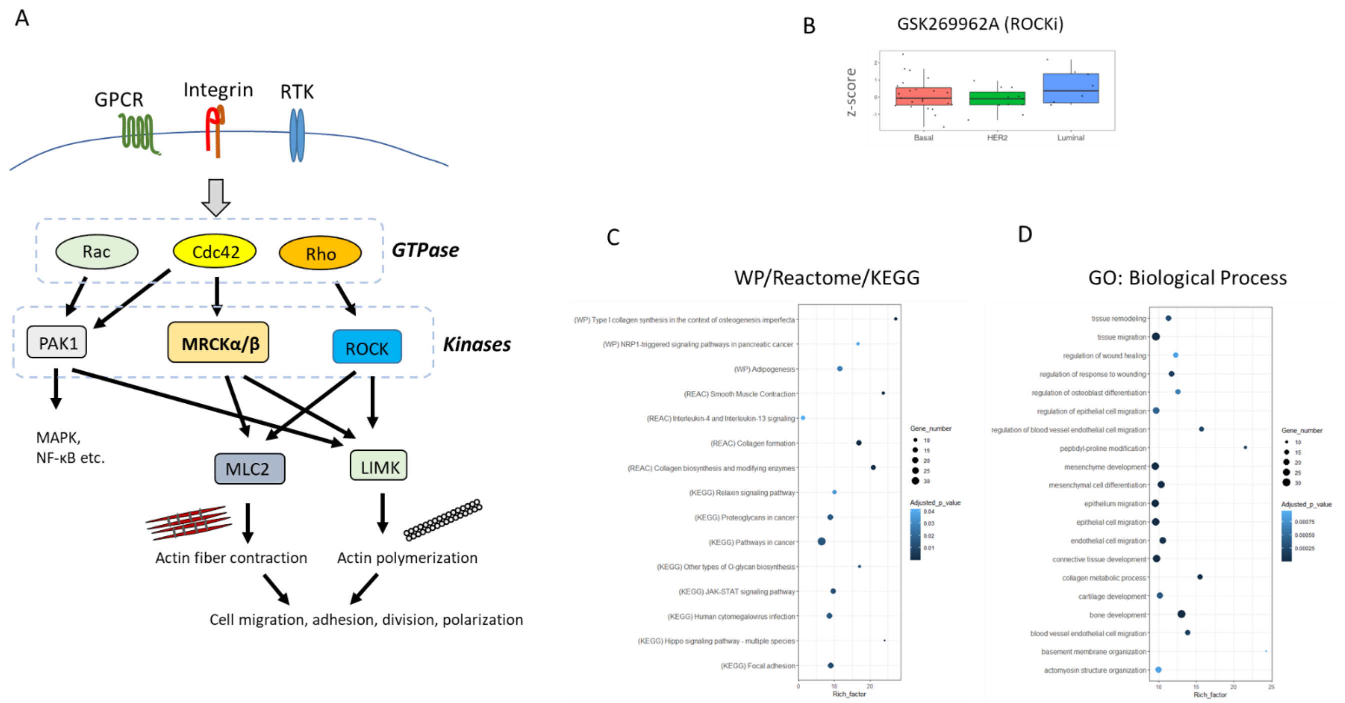

Supplementary Figure S2

(A) Schematic drawing of the pathways of Rho GTPases and downstream effector kinases. Rho GTPase family includes Rac, Cdc42 and Rho, which are activated by various GPCRs, integrins and RTKs. Activated Rho GTPases activates downstream kinases, such as PAC1, MRCK, and ROCK. MRCK and ROCK have several common targets and both of them regulates actin polymerization and actin fiber contraction. **(B)** Boxplot with jetter showing IC50 of ROCK inhibitor GSK269962A in the breast cancer cells used in Fig 1. **(C and D)** The dot plots showing the results of pathway analysis using the upregulated genes in the sensitive cell lines compared to the resistant cell lines. The result using the WikiPathway, Reactome and KEGG datasets **(C)** and Gene Ontology (Biological Process) **(D)**.

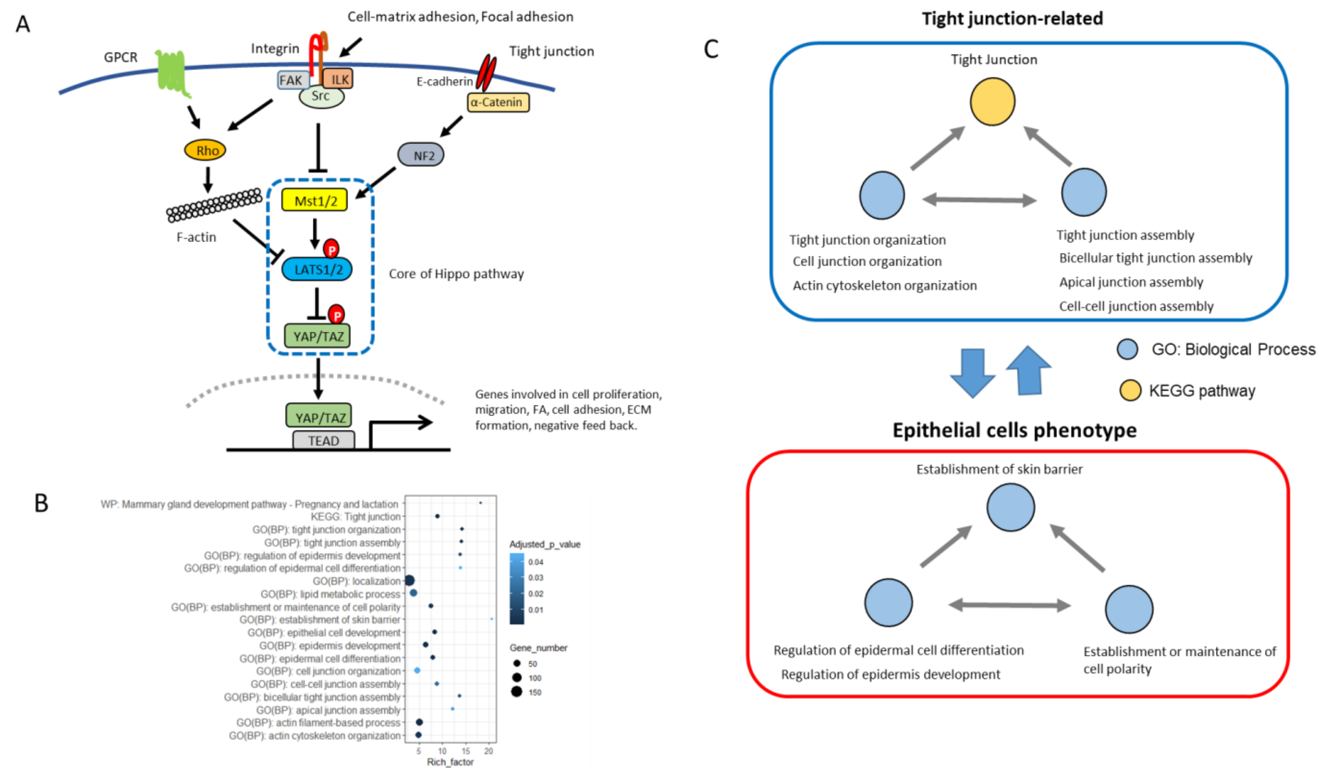

Supplementary Figure S3

(A) Schematic drawing of the Hippo signaling pathway. The core molecules of the Hippo pathway are two kinases, MST and LATS, and the transcription co-regulator YAP/TAZ. MST activates LATS by phosphorylating it, and LATS represses it by phosphorylating YAP/TAZ. Unphosphorylated YAP/TAZ binds to the transcription factor TEAD and induces transcription of many target genes including *CCN1*, *CCN2*, *MYC* and *CCND1*. YAP/TAZ promotes various cellular functions such as cell proliferation, migration, and stem cell function. YAP/TAZ also promotes extracellular matrix production and focal adhesion formation. On the other hand, MST and LATS are regulated by various upstream signals. Signals from tight junctions activate MST/LATS and repress YAP/TAZ. In contrast, Signals from integrin and GPCRs suppress LATS through RhoA-mediated F-actin formation, resulting in activation of YAP/TAZ. **(B)** Pathway analysis was performed using genes that express higher in the resistant cell lines than the sensitive cells. **(C)** A summary of the results of pathway analysis in **(B)**.

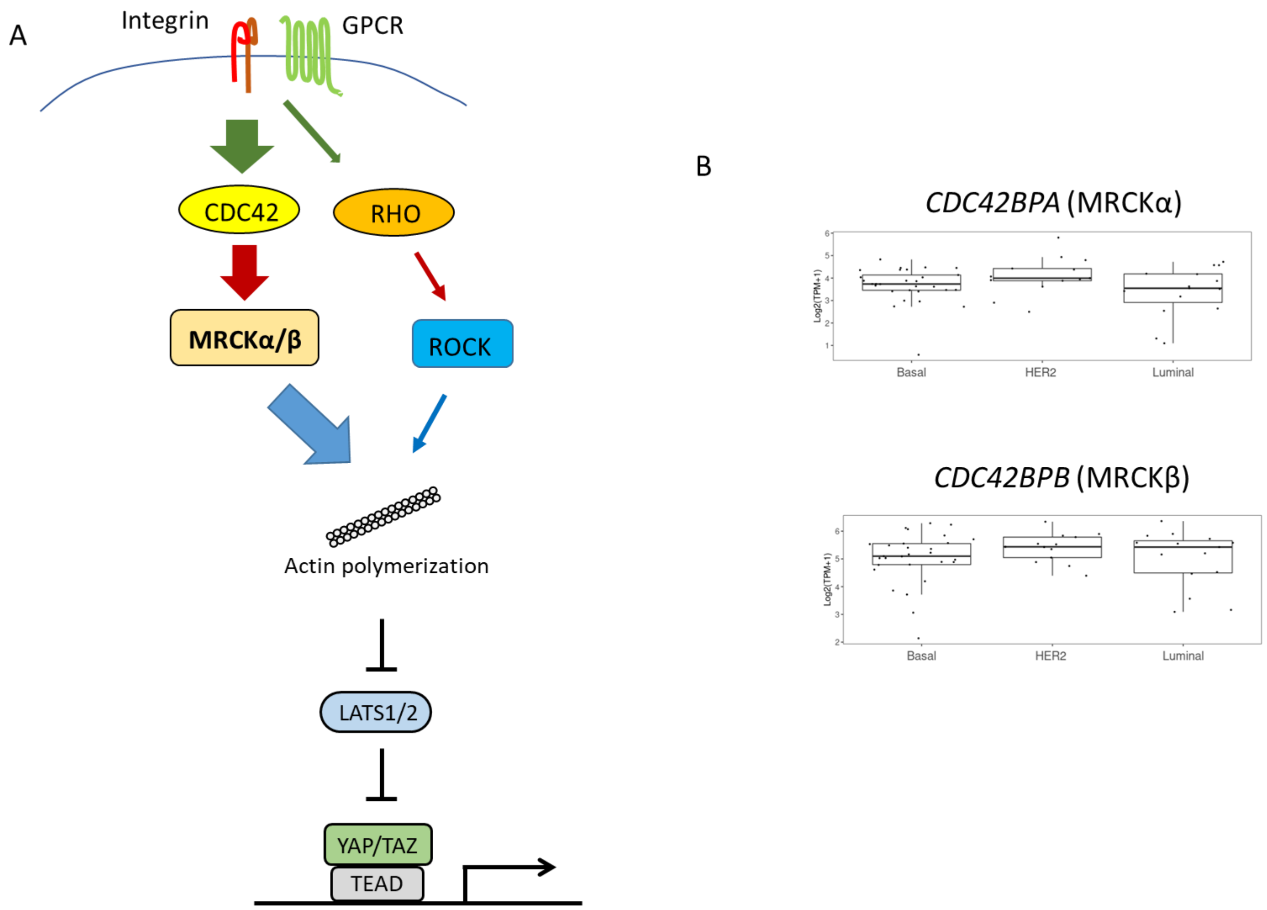

Supplementary Figure S4

(A) The hypothesis of the MRCK-mediated YAP/TAZ regulation in TNBC. It is well established that YAP/TAZ is activated via Integrin/GPCR - RhoA - ROCK - F-actin. In some TNBC cells, however, Cdc42-MRCK pathway plays a dominant role for the regulation of F-actin formation. Moreover, YAP/TAZ plays a more critical role for cell survival and proliferation in TNBC than other subtypes. Therefore, the inhibition of MRCK most effectively suppress TNBC in all subtypes of breast cancer. **(B)** The *CDC42BPA* and *CDC42BPB* expression in the breast cancer cell lines used for the screening in Fig. 1 is shown in the boxplots with jetters by subtype.

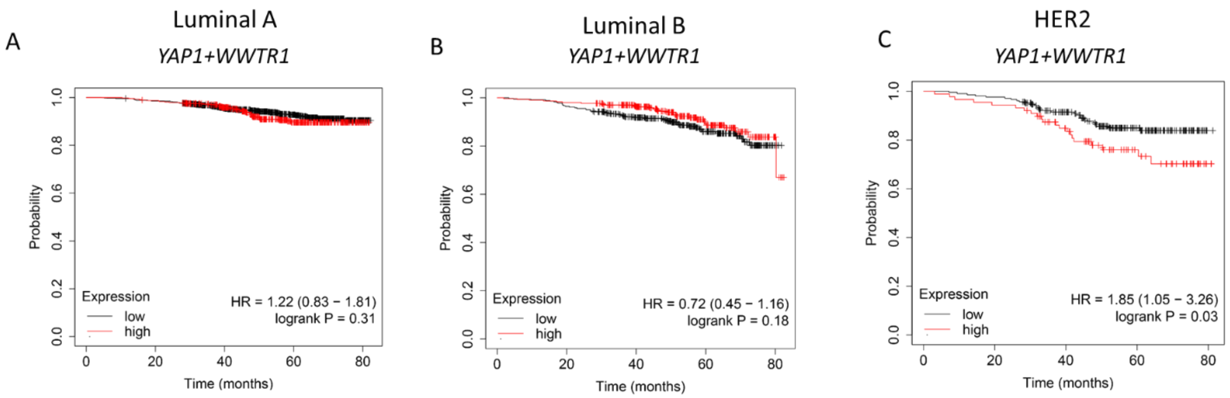

Supplementary Figure S5

Breast cancer patients with different subtypes were divided into two groups based on the high and low total *YAP1/WWTR1* expression levels, and their survival curves were compared. Luminal A **(A)**, Luminal B **(B)**, and HER2 **(C)** subtype breast cancer (Logrank P=0.31, 018 and 0.03, respectively).

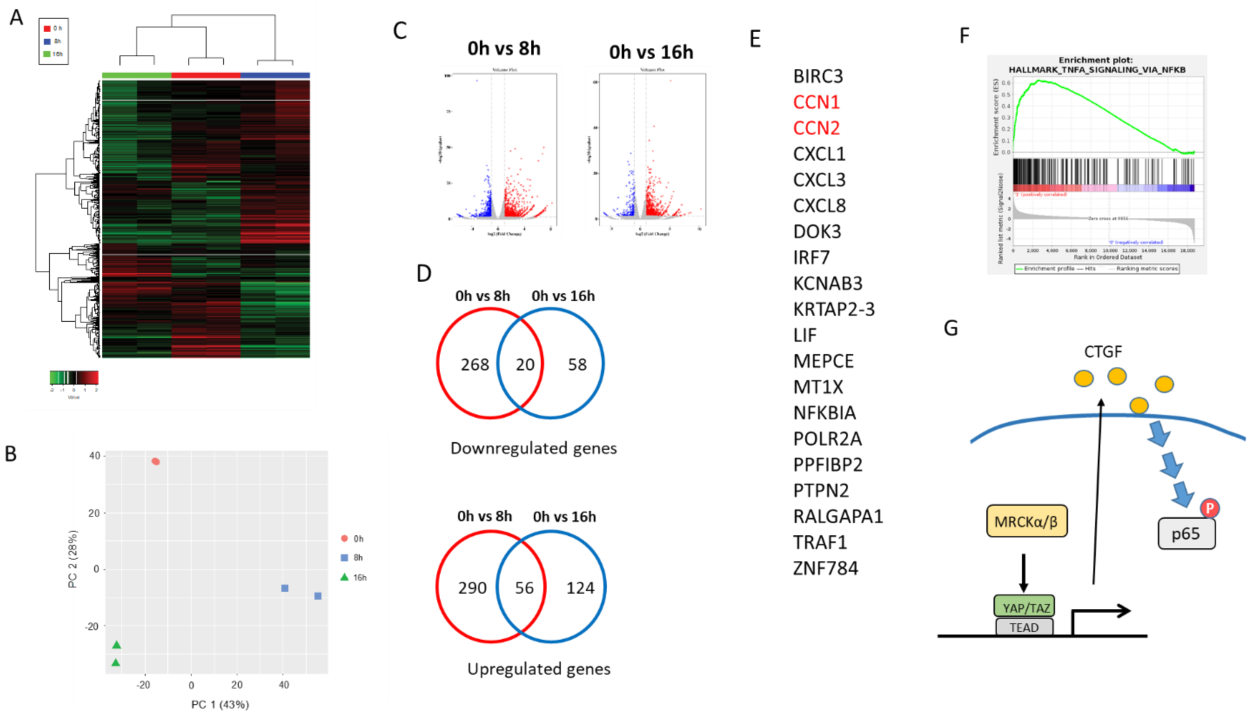

Supplementary Figure S6

(A) BT549 cells were treated/untreated with BDP-9066 for 8 and 16 hours and subjected to RNA sequencing analysis. Hierarchical clustering by differentially expressed genes were performed. **(B)** Principal component analysis was performed with the gene expression profiles of BT549 cells BDP-9066 used in **(A)**. **(C)** Volcano plots representing differentially expressed genes between control and BDP-9066-treated cells. **(D)** The upregulated and downregulated genes are shown in venn diagram. **(E)** The list of genes downregulated in both 8 and 16 hours samples compared to the untreated control. **(F)** Gene Set Enrichment Analysis with the hallmark gene sets was performed using the gene expression profiles of the resistant and sensitive cell lines used in Fig 2. HALLMARK_TNFA_SIGNALING_VIA_NFKB shows the third highest enrichment score in the sensitive cell lines. Normalized Enrichment score is 1.54 and Nominal p-value is 0.0. **(G)** Schematic drawing of the MRCK-YAP/TAZ-CTGF-NF-κB pathway. MRCK activates YAP/TAZ and promotes the production of its target CTGF. CTGF acts on cells via various receptors, resulting in activation of NF-κB. How CTGF activates NF-κB is still poorly understood.

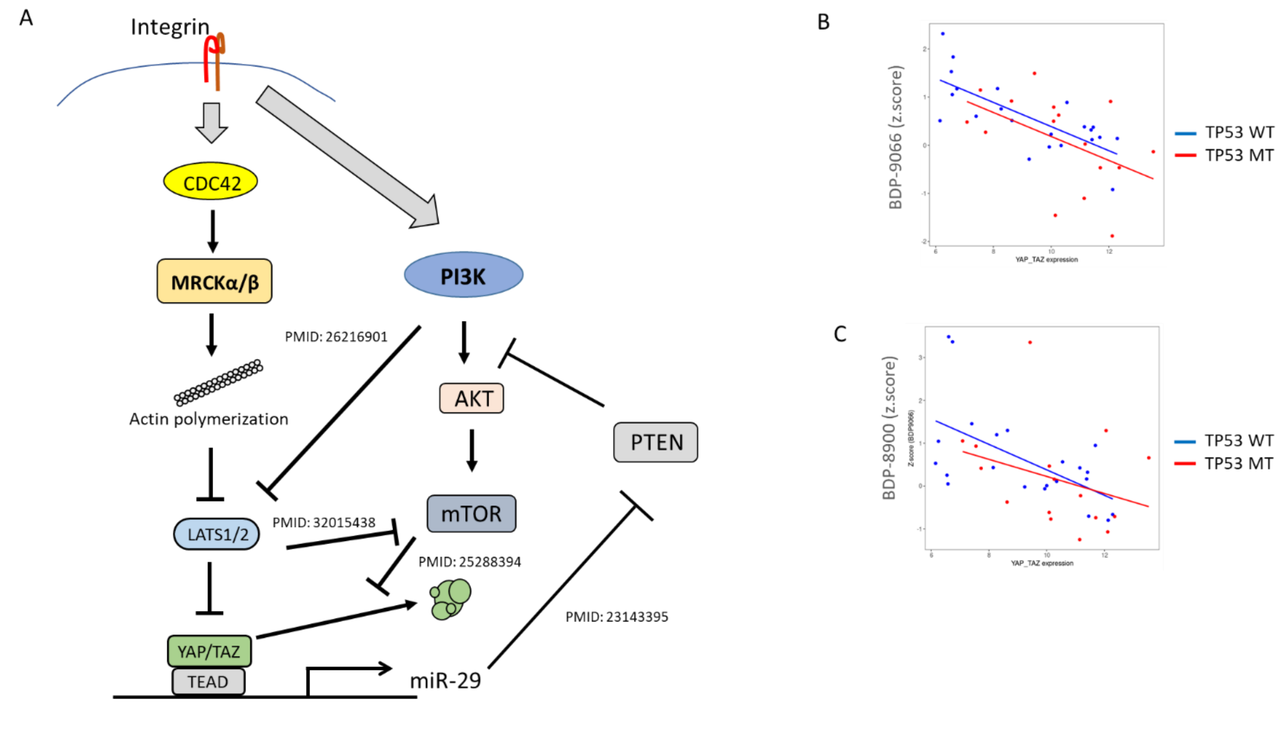

Supplementary Figure S7

(A) Schematic drawing of the crosstalk between Hippo signaling and PI3K-AKT pathways. YAP/TAZ activates the PI3K-AKT pathway by downregulating PTEN, while several studies show that the PI3K-AKT pathway activates YAP/TAZ by inhibiting upstream kinases or YAP/TAZ degradation. **(B and C)** The relationship between *YAP1/WWTR1* expression and sensitivity to MRCKi in breast cancer cell lines is shown in scatter plots using regression lines. TP53 mutant cells are indicated by red dots, TP53 wild-type cells are indicated by blue dots, and their respective regression lines are also indicated by the same color. *YAP1/WWTR1* expression on the X-axis and IC50 against BDP-9066 **(B)** or BDP-8900 **(C)** on the Y-axis.
